# Taste Sensitivity and Taste Preference among Malay Children Aged 7 to 12 Years in Kuala Lumpur—A Pilot Study

**DOI:** 10.3390/pediatric13020034

**Published:** 2021-05-18

**Authors:** Ler Sheang Lim, Xian Hui Tang, Wai Yew Yang, Shu Hwa Ong, Nenad Naumovski, Rati Jani

**Affiliations:** 1 Division of Nutrition and Dietetics, School of Health Sciences, Faculty of Medicine and Health, International Medical University, Kuala Lumpur 57000, Malaysia; LIM.LERSHEANG@student.imu.edu.my (L.S.L.); TANG.XIANHUI@student.imu.edu.my (X.H.T.); OngShuHwa@imu.edu.my (S.H.O.); 2 Discipline of Nutrition and Dietetics, School of Rehabilitation and Exercise Sciences, Faculty of Health, University of Canberra, Canberra, ACT 2617, Australia; Nenad.naumovski@canberra.edu.au (N.N.); Rati.Jani@canberra.edu.au (R.J.); 3 Functional Foods and Nutrition Research (FFNR) Laboratory, University of Canberra, Bruce, ACT 2617, Australia

**Keywords:** children, body weight status, taste sensitivity and preferences

## Abstract

The taste and food preferences in children can affect their food intake and body weight. Bitter and sweet taste sensitivities were identified as primary taste contributors to children’s preference for consuming various foods. This pilot study aimed to determine the taste sensitivity and preference for bitter and sweet tastes in a sample of Malaysian children. A case–control study was conducted among 15 pairs of Malay children aged 7 to 12 years. Seven solutions at different concentrations of 6-n-propylthiouracil and sucrose were prepared for testing bitterness and sweet sensitivity, respectively. The intensity of both bitter and sweet sensitivity was measured using a 100 mm Labelled Magnitude Scale (LMS), while the taste preference was rated using a 5-point Likert scale. The participants were better at identifying bitter than sweet taste (median score 6/7 vs. 4/7). No significant differences were detected for both tastes between normal-weight and overweight groups (bitter: 350 vs. 413, *p* = 0.273; sweet: 154 vs. 263, *p* = 0.068), as well as in Likert readings (bitter 9 vs. 8: *p* = 0.490; sweet 22 vs. 22: *p* = 0.677). In this sample of Malay children, the participants were more sensitive to bitterness than sweetness, yet presented similar taste sensitivity and preference irrespective of their weight status. Future studies using whole food samples are warranted to better characterize potential taste sensitivity and preference in children.

## 1. Introduction

The increase in rates of childhood obesity has become a worldwide health problem. Globally, over 340 million children and adolescents aged 5 to 19 years were overweight or obese in 2016 [1]. According to the National Health and Morbidity Survey (NHMS IV 2019), 29.8% of Malaysian children in a similar age group are overweight (15.0%) and obese (14.8%) [2], making Malaysia one of the Asian countries with the highest percentage of childhood obesity [3]. Furthermore, several studies have demonstrated that childhood overweight and obese status can pose significant health risks later in life, increasing risk factors associated with cardiovascular diseases, type 2 diabetes mellitus and hypercholesterolemia [3,4]. Disruption in energy balance is also an important contributory factor due to increased sedentary lifestyle and intake of high-energy food [5,6,7].

Children’s food preferences are learnt innately and could drive the overconsumption of specific foods, especially foods with a high sugar content [5,8]. Therefore, taste sensitivity plays an important and unavoidable role in food preferences, choices, and consumption [9]. Taste is described as one of the most important neuronal sensations for the evaluation of food content, consequently resulting in food selection and potentially influencing the overall food intake. It also plays a primal function in determining food as nutritious or noxious and in identifying potentially toxic substances, consequently preventing their ingestion [10,11]. In humans, the traditional sensation of taste is described using the five basic modalities defined as ‘salty’, ‘sweet’, ‘sour’, ‘bitter’ and ‘umami’, with the last one being a relatively new descriptor commonly ascribed to savory or pleasant food [10]. The bitter taste is commonly ascribed to some polyphenolic compounds usually found in certain green vegetables [12,13]. Specific taste sensitivity and perception towards bitterness were reported to heighten the preference for sweetness, resulting in increased consumption of sweet foods in children [14,15].

The perception of taste is quite commonly confused with the sensation of flavor, which derives from the combination of different chemo-neuronal mechanisms involving olfaction, texture, temperature and taste itself, all depending on efficient saliva production [10,11,16]. It is well known that sensitivity to n-propylthiouracil (PROP) is the best-known example of taste variability that has broad implications for taste perception, food preferences and dietary behavior, with subsequent impacts on nutritional status and health outcome [17]. Based on PROP sensitivity, individuals can be classified in three PROP taster categories: non-tasters (who are taste-blind to the PROP compound), medium tasters, and PROP super-tasters (who perceive extreme bitterness when tasting PROP) [18]. A recent study amongst 156 Caucasians and 67 Asians aged 18–65 years in UK reported a higher proportion of Asians were super-taster as compared to Caucasians (55% vs. 24%, *p* < 0.0001) [19]. Furthermore, it is well established that super-tasters are more responsive to other taste qualities including fats [20] and that PROP tasting is associated with variations in food acceptability, selection of vegetables and fruits and several health parameters, such as body weight status in children, and secondary to colorectal cancer risk in older adults being linked to inadequate vegetable intake [21,22,23,24,25].

Taste sensitivity differs individually based on age, sex, ethnicity, body weight status or body mass index (BMI), taste bud development, taste concentration and saliva composition [15,26,27,28,29,30,31]. A relatively recent review investigating the relationship between bitter taste sensitivity of children and weight status has identified two out eight studies that reported a higher BMI status in children displaying a greater perception of the bitter taste [32]. The Malay ethnicity regards 61.8% of the Malaysian population [33], and one-third of obese individuals in Malaysia are Malay [34]; hence, the Malay population needs to be investigated in a targeted way [35,36]. Therefore, the aim of this study was to compare taste sensitivity and preference between overweight and normal-weight (as determined by BMI) primary school-aged Malay children for sweet and bitter tastes.

## 2. Materials and Methods

Ethics approval of the study [BDN I-2019 (20)] was obtained from the research and ethics committee of the International Medical University. Informed written consent was provided by the parents of children who participated in the study, alongside with children assent. Socio-demographics information including age, gender, parental educational level, employment status and family’s household income was also collected. Ethnicity was defined by the official registration of race in the child’s birth certificate. The study was conducted in well-ventilated rooms at local primary schools (*n* = 2) and communities (*n* = 2) from February to March 2020.

**Figure 1:**
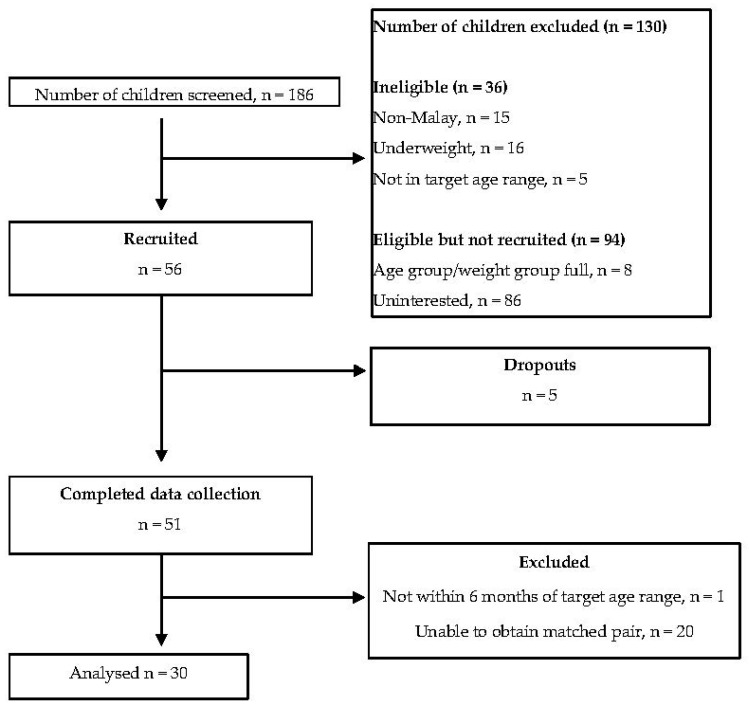
Flow chart indicating screening, recruitment, data collection and data analysis.

A matched pair case–control study was conducted amongst Malay children in Klang Valley area, Malaysia. Using a specific formula to calculate differences in two populations [37] and the standard deviation reported from a similar study by Virgini et al. investigating taste sensitivity and BMI [31], the mean proportion of correct identification between normal weight and overweight was 0.15 (SD 0.15); therefore, the estimated sample size was 16 children per group. However, due to the global Covid-19 pandemic during the study period, the study ceased at a sample size of 15 children per group. The eligible criteria included Malay children aged 7 to 12 years with parental consent and good health conditions. Those children who were underweight, taking medication for chronic illness such as thyroid illness, nasal and oral infections, diabetes and other non-communicable diseases in the past three months, indulging in substance abuse including smoking, drugs, alcohol or any form of addiction were excluded from the study (Figure 1). Participants were matched within a 12-month age difference (+/− 6 months old) and for gender in the normo-weight and overweight groups. World Health Organization (WHO) growth charts were used for the classification of overweight (BMI-for-age Z-score +1 SD and above) and normal (BMI-for-age Z-score between 0 and <+ 1 SD) [38]. Body weight was determined using a calibrated weighing scale (TANITA HD-314, Tanita Corporation, Tokyo, Japan) to the nearest 0.1 kg, while height was measured using a portable stadiometer (SECA 213, Seca Group, Hamburg, Germany) to the nearest 0.1 cm.

### 2.1. Taste Sensitivity and Preference Testing

Two basic taste qualities, bitterness and sweetness, were tested in the participants. The 6-n-propylthiouracil (Sigma Aldrich, Castle Hill, Australia) was used in this study to prepare six water solutions at different concentrations for bitterness testing (PROP concentrations: 0.017, 0.056, 0.180, 0.560, 1.80, 3.20 mmol/L). The PROP solution is reported to be a safe solution that is valid and suitable to be used in sensory evaluation studies with children [32]. The bitterness concentration was adopted from Jani et al. protocol (2020) investigating bitter taste sensitivity in Australian children aged 7 to 12 years [39]. The sweetness stimulus consisted of six water solutions of sucrose (sucrose concentrations: 0.263, 0.646, 2.375, 7.128, 21.385, 48.613 mmol/L). The sweetness concentration was selected in consideration of the children’s taste detection and recognition threshold [40]. Therefore, the highest concentration was expected to be correctly identified by almost 100% of the participants [40]. All taste solutions were prepared, cups were pre-numbered manually and labelled in random coding to carry out a blind experiment for both participants and interviewers. Participants were presented with 14 cups in increasing concentration order of sweet and bitter stimuli.

Participants were asked to rate their perceived intensity of the stimuli using a well-validated Labelled Magnitude Scale (LMS) with a rating from 0 to 100. The LMS response equal to zero indicated that bitterness or sweetness was ‘barely detectable’, whereas a response of 100 indicated ‘strongest imaginable’. The perceived intensity of stimuli reflects the taste sensitivity of participants. For taste preference, participants were asked to rate their preference using a 5-point Likert scale, 1 corresponding to ‘Least preferred’ and 5 to ‘Most preferred’. The Likert scale was presented using emoticons for improving the comprehension of participants of taste preference rating [41].

### 2.2. Procedures

The participants were instructed to abstain from eating or drinking, except for water, for at least one hour before the test. Furthermore, before testing, the participants were asked to determine which foods they perceived as bitter and sweet using sets of food photographs. This step ascertained the participants’ knowledge towards taste quality [30]. The testing was conducted in a non-forced choice paradigm due to its advantage to assess taste detection and identification in individual participant [42]. During the test, each participant was tested with 14 solutions, as two water solutions served as blanks (no taste), and 6 concentrations of sweet and bitter. Testing started with the lowest concentrations, and the order of presentation was reproducible by the researchers (LSL and XHT). The participants were presented with 2 ml solutions in small cups at room temperature (24 °C). In addition, a spitting cup and a cup of plain water to be used as a mouth rinse and tissue between the samples were provided. A story game was created about a magician who had prepared 14 magic solutions into small cups, to firstly ask the participant to identify the respective taste [43]. They were informed to take a sip from each testing solution presented and spit out into the cup provided. Then, the participants were asked to identify the taste intensity of each “magic” solution by marking a cross (X) on a paper reporting the LMS. Lastly, the participants rated their preference towards each solution using a 5-point Likert scale [39]. Between each solution at approximately 2 min of interval, the participants were advised to rinse their mouth and have a plain cracker (Meiji^®^, Meiji Seika, Singapore) to minimize taste fatigue.

### 2.3. Statistical Analysis

Statistical Package for Social Sciences (SPSS, version 25, IBM, New York, NY, USA) was used for all analyses in the study. Normality was tested by the Shapiro–Wilks test, normally distributed data are presented as mean ± standard deviation, while median (interquartile range) was determined for non-normally distributed data. The Mann–Whitney *U*-test was selected to compare the differences in taste sensitivity and taste preference between normal-weight and overweight groups. The Spearman’s rho correlation test was used to determine the relationship between taste sensitivity and taste preference for each concentration of PROP and sucrose, respectively. A partial correlation test was carried out to study the relationship between taste sensitivity and taste preference when controlling for family monthly household income, age, gender, and weight status as covariates. The relationship between weight status and taste sensitivity was examined using repeated measures ANOVA for PROP and sucrose solutions, and a probability value of *p* < 0.05 was considered significant.

## 3. Results

A total of 51 participants were recruited in the study. After match-pairing, data from 15 pairs of participants (*n* = 30) were used for data analysis and reporting in the study. Mean age of the participants was 10.1 ± SD 1.6 years. The study comprised more females (*n* = 18) than males (*n* = 12) participants. As expected, weight status was significantly different between normal-weight and overweight groups. Parental educational level and household income were similar between the groups. Almost all children were from low-income families (Table 1).

**Figure 2:**
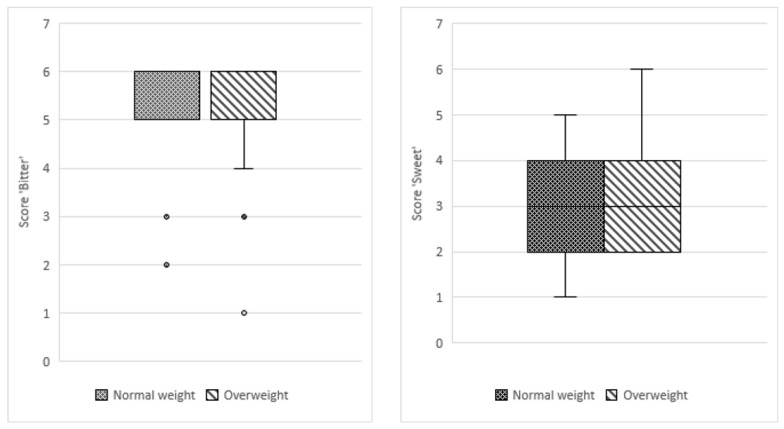
Boxplot of median taste identification scores for bitter and sweet between normal-weight and overweight groups.

The participants had more difficulties in correctly identifying the sucrose solution compared to the PROP solution, resulting in a lower score, although sucrose concentration in solution, median score: 209.5 (IQR 201.5) was much higher than that of PROP, median score: 367.5 (IQR 250). For both taste qualities, more participants in the overweight group were able to make a correct identification as compared to participants in the normalweight group (bitter: 78 vs. 75; sweet: 49 vs. 44). Both groups obtained the same median score in identifying bitter (score of 6) and sweet (score of 4) tastes in the prepared solutions including plain RO water (blank). No significant differences between the study groups were observed in taste identification for PROP (*p* = 0.530) and sucrose solutions (*p* = 0.460; Figure 2).

**Figure 3:**
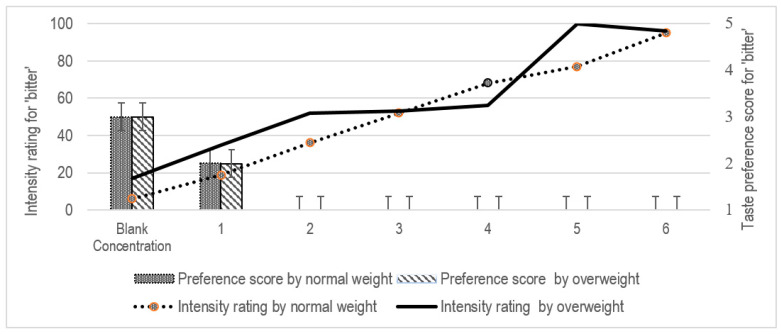
Intensity rating and taste preference for ‘bitter’ between the study groups.

Overall, no significant differences in taste preference for both stimuli (sweet and bitter) were observed between the study groups for each concentration (*p* > 0.05 in all cases) (Table 2). When determining taste intensity using the LMS scale, a higher score indicated that a stronger taste intensity was perceived, with a maximum score of 100. No significant differences between the study groups were observed in rating the concentrations of PROP solutions (*p* > 0.05 in all cases) (Figure 3). An inverse relationship between bitter taste intensity and preference scores was observed in both groups, wherein a higher intensity rating was associated with a lower preference score (Table 3). Both groups disliked the PROP solution at concentrations beyond Concentration 2.

**Figure 4:**
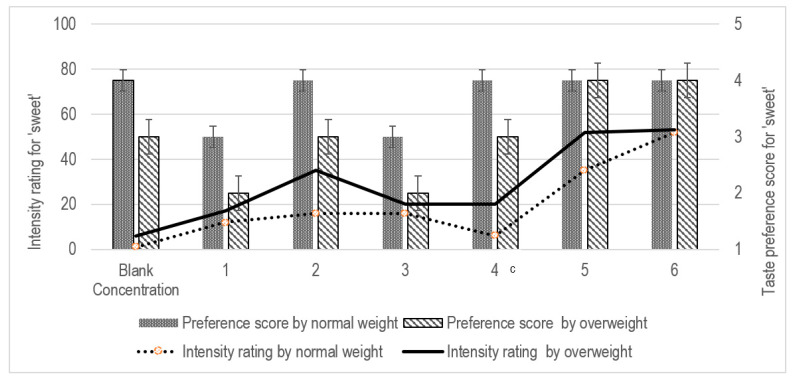
Intensity rating and taste preference for ‘sweet’ between the study groups. The Spearman correlation test was used, *p*-value < 0.05 ^c^.

For the sucrose solutions, the overweight group indicated a significant higher intensity for Concentration 4 compared to the normal-weight group (LMS score of 20 vs. 6, *p* = 0.024; Figure 4). These participants had higher preference towards sucrose solutions t higher concentrations (Median preference score of 4 at Concentration 4 vs. score of 3 at lower concentrations, *p* > 0.05; Table 3).

The results presented in Table 3 indicate the Spearman’s rho correlation between participants’ taste sensitivity and taste preference. Higher taste sensitivity reduced participant’s taste preference significantly (r = −0.538, *p* = 0.002) when PROP concentration corresponded to Concentration 4 (0.56 mmol/L). For the sucrose solution, no significant correlation between taste sensitivity and taste preference was detected in all participants (Table 3, *p* > 0.05 for each concentration of sucrose solution). Comparisons within the groups, indicated that concentrations of PROP solution corresponding to 3 (r = −0.568, *p* = 0.027) and 4 (r = −0.541, *p* = 0.037) negatively affected the taste preference in the normal-weight but not in the overweight group (Table 3). Interestingly, taste preference was strongly correlated with the sucrose solution offered at Concentrations 1 to 3 for the overweight participants. At Concentrations 4 and beyond in this study, both stimuli appeared to reach a saturation level, interfering with detection by the participants (Table 2).

There was no association between taste sensitivity and food preference between the study groups after controlling for age, gender and socio-economic status; F(1, 28) = 0.965, *p* = 0.334 for bitterness; F(1, 28) = 3.823, *p* = 0.061 for sweetness. Consistent with the findings from the bivariate correlation, when controlling for socio-economic status, age, gender and weight status as covariates using a partial correlation coefficient, taste preference was found to be significantly correlated with taste sensitivity at Concentrations 3 (r = −0.410, *p* = 0.033) and 4 (r = −0.561, *p* = 0.003) of PROP solutions. For the sucrose solution, the controlling of covariates did not demonstrate significant correlation between taste preference and taste sensitivity of the participants, except at Concentration 2 (r = 0.465, *p* = 0.017).

## 4. Discussion

To the best of our knowledge, this exploratory study is the first local study investigating taste sensitivity using PROP and sucrose solutions in normal-weight and overweight Malay children aged 7 to 12 years. The findings add new knowledge for understanding Asian Malay children’s sensitivity towards bitterness and sweet taste; however, no statistical differences were detected in taste sensitivity and preferences between normal-weight and overweight children.

Malaysian children are proposed to be frequently exposed to a strong flavored and spicy cuisine. [44] However, finding from this study indicate that their taste perception might be similar to that of other children. In a study investigating Caucasian children, it was reported that those children generally have a higher sensitivity for bitterness [45]. Findings indicate that Malay children have an innate preference towards sweetness and reject bitterness [14,15,46], with a strong potential towards consumption of energy-dense food (mainly tasting ‘sweet’) and avoidance of bitter-taste food (green vegetables) [46]. Children with a higher acceptance and exposure to sweet foods demonstrated a lower sensitivity for sweet [47,48]. Generally, those children are more sensitive to bitterness and less sensitive to sweetness [45,47,48].

The findings of this study also demonstrated a higher sensitivity towards bitterness in our Malay children population sample compared to Korean children from examined by Chung et al. [40], who showed a recognition threshold at 0.14 g/L. The participants strongly disliked the bitter solution even at Concentration 1, corresponding to 0.0029 g/L. This could be due to prior exposure and experience of food that could impact the bitter preference development in children. Furthermore, this could also result in children disliking bitterness if they had not been frequently exposed to bitter-tasting food [49], in contrast to a higher exposure to sweet-tasting food [5,8]. In this study, children’s prior exposure towards bitterness and sweetness was not investigated, which could possibly provide more in-depth information to the body of knowledge on taste in children.

The rating of the taste intensity at Concentration 3 for bitterness and sweetness in all groups of children was lower than the one at Concentrations 2 and 1. Both bitter and sweet receptors are G-protein-coupled receptors (GPCRs), which were first identified in taste bud type II cells. [50] Taste receptor family 1 subtype 2 and 3 (T1R2/T1R3) is known to respond to sweetness, while the bitter taste receptor is mainly the taste receptor 2 (T2Rs) [50]. Since both bitterness and sweetness ligands need to bind to their respective GPCRs to trigger a taste sensation, the sensitivity of taste may be disrupted if the children did not rinse their mouth fully, as these residue might remain on taste buds. This might also provide a potential explanation of the observation in our study that the children perceived lower intensity for bitterness and sweetness at Concentration 3 than at Concentrations 2 and 1. Nevertheless, this study protocol included a relatively low number of solutions presented to the children within 20 min. The study time took into consideration the attention time of children aged 7 to 12 years, as the normal attention span of children is estimated between 3 to 5 min per year of child’s age [51].

No significant difference was observed in the perceived taste intensity rating between normo-weight and overweight groups. This finding is in line with the systematic review by Tepper et al. [7], which reported no association between tasting ability of PROP and sweet in food products and the body weight of children. In contrast, other studies reported children with higher BMI had a reduction in taste sensitivity [6,31,52]. This study controlled for sex, age and ethnicity, which were identified to be dependent factors influencing taste sensitivity in children.

### Limitations

This case–control study could not determine the cause–effect relationship between taste sensitivity, taste preference and weight status of children, as data were collected at a single time point. PROP might be too bitter to test the preference for bitter and not represent normal bitter-tasting foods [53]. The small sample size in the present study could be a limiting factor; hence, future studies involving other regions and ethnicities in Malaysia are warranted. This study, however, did not assess children’s usual food avoidance or dietary intake and its association with taste sensitivity. The preliminary results call for more research to ascertain the relationship between taste sensitivity of five basic tastes (bitter, sweet, sour, salty and umami) and food consumption of local children.

## 5. Conclusions

Taste sensitivity and preference were similar between normal-weight and overweight children in this study. Perceived intensity of bitter taste was inversely correlated with taste preference; in contrast, for the sweet taste, we observed increasing liking with higher concentrations of sucrose. In future studies of the relationship between food preference and taste quality, it is recommended to use a whole food sample with bitter taste in replacement of the PROP solution, such as bitter melon (*Momordica charantia)* or bitter bean (*Parkia speciose*). Similarly, children’s avoidance and acceptance of food can be determined to provide better insights. Practical strategies could subsequently be developed for the prevention and management of childhood obesity by considering children’s taste sensitivity and food preference.

## Figures and Tables

**Table 1:** Characteristics of the participants (*n* = 30).

	All (*n* = 30)	Normal Weight (*n* = 15)	Overweight (*n* = 15)	*p*-Value
Age (years), mean ± SD	10.13 ± 1.57	10.13 ± 1.69	10.13 ± 1.51	0.600 ^a^
Gender, *n* (%)				1.000 ^b^
Male	12 (40)	6 (50)	6 (50)	
Female	18 (60)	9 (50)	9 (50)	
Anthropometry, mean ± SD				
Weight, kg	35.70 ± 13.11	27.52 ± 8.47	43.87 ± 11.87	< 0.001 ^a^
Height, cm	135.28 ± 12.62	130.51 ± 11.88	140.05 ± 11.83	0.036 ^a^
BMI, kg/m^2^	18.89 ± 3.99	15.83 ± 2.10	21.94 ± 2.92	< 0.001 ^a^
Father’s education, *n* (%)				0.054
No formal	1 (3.3)	1	0	
Primary	5 (16.7)	4	1	
Secondary	20 (66.7)	9	11	
Tertiary	4 (13.3)	1	3	
Mother’s education, *n* (%)				1
No formal	0	0	0	
Primary	4	2	2	
Secondary	20	10	10	
Tertiary	6	3	3	
Family household income (RM), *n* (%)				0.153
<4500	28	15	13	
4501–10,000	2	0	2	
>10,000	0	0	0	

^a^ Independent sample *T*-test was used, *p*-value < 0.05. ^b^ Chi-square test was used, *p*-value < 0.05.

**Table 2:** Comparison between median taste sensitivity score and food preference score between the study groups.

	Normal Weight (*n* = 15)	Overweight/Obese (*n* = 15)	*p*-Value	Normal Weight (*n* = 15)	Overweight/Obese (*n* = 15)	*p*-Value
	Taste Intensity Rating, Median (IQR)			Taste Preference, Median		
Bitter (PROP Solution)						
Concentration 1	19 (36)	35 (43)	0.189	2	2	0.713
Concentration 2	36 (42)	52 (39)	0.647	0	0	0.902
Concentration 3	52 (75)	53 (47)	0.189	0	0	0.512
Concentration 4	68 (81)	56 (47)	0.441	0	0	0.567
Concentration 5	77 (67)	100 (12)	0.075	0	0	0.775
Concentration 6	95 (64)	96 (48)	0.686	0	0	0.870
Sweet (Sucrose solution)						
Concentration 1	12 (18)	17 (79)	0.183	3	2	0.267
Concentration 2	16 (46)	35 (36)	0.158	4	3	0.539
Concentration 3	16 (15)	20 (37)	0.055	3	4	0.595
Concentration 4	6 (34)	20 (79)	0.024 *	4	3	0.367
Concentration 5	35 (35)	52 (78)	0.117	4	4	0.595
Concentration 6	52 (60)	53 (82)	0.502	4	4	1.000

* Mann–Whitney U-test was used, *p*-value < 0.05.

**Table 3:** Spearman’s rho correlation for taste sensitivity and taste preference.

Taste	Solute Concentration (mmol/L)	Spearman’s Rho Correlation (r), *p*-Value		
		Overall (*n* = 30)	Normal Weight (*n* = 15)	Overweight (*n* = 15)
Bitter (PROP solution))				
Concentration 1	0.017	−0.220, 0.243	−0.285, 0.304	−0.179, 0.523
Concentration 2	0.056	−0.129, 0.497	−0.297, 0.283	−0.113, 0.687
Concentration 3	0.18	−0.504, 0.005	−0.568, 0.027	−0.295, 0.286
Concentration 4	0.56	−0.538, 0.002	−0.541, 0.037	−0.232, 0.406
Concentration 5	1.80	−0.215, 0.255	−0.111, 0.694	−0.180, 0.520
Concentration 6	3.20	−0.257, 0.171	−0.459, 0.085	−0.137, 0.627
Sweet (Sucrose solution)				
Concentration 1	0.263	0.155, 0.413	−0.162, 0.564	0.556, 0.032
Concentration 2	0.646	0.292, 0.118	0.133, 0.636	0.623, 0.013
Concentration 3	2.375	0.204, 0.279	0.019, 0.948	0.541, 0.037
Concentration 4	7.128	−0.095, 0.617	0.079, 0.781	−0.243, 0.383
Concentration 5	21.385	0.186, 0.326	0.167, 0.551	0.161, 0.567
Concentration 6	48.613	0.216, 0.253	0.162, 0.565	0.289, 0.296

## Data Availability

The data presented in this study are available on request from the corresponding author. The data are not publicly available due to confidentiality.
